# Recapitulation of the Function and Role of ROS Generated in Response to Heat Stress in Plants

**DOI:** 10.3390/plants10020371

**Published:** 2021-02-15

**Authors:** Emily Medina, Su-Hwa Kim, Miriam Yun, Won-Gyu Choi

**Affiliations:** 1 Department of Biochemistry and Molecular Biology, University of Nevada, Reno, NV 89557, USA; emilymedina@nevada.unr.edu (E.M.); suhwakim@unr.edu (S.-H.K.); 2 Biology and Psychology Department, University of Nevada, Reno, NV 89557, USA; myun@nevada.unr.edu

**Keywords:** reactive oxygen species (ROS), ROS signals, heat stress, stress combination

## Abstract

In natural ecosystems, plants are constantly exposed to changes in their surroundings as they grow, caused by a lifestyle that requires them to live where their seeds fall. Thus, plants strive to adapt and respond to changes in their exposed environment that change every moment. Heat stress that naturally occurs when plants grow in the summer or a tropical area adversely affects plants’ growth and poses a risk to plant development. When plants are subjected to heat stress, they recognize heat stress and respond using highly complex intracellular signaling systems such as reactive oxygen species (ROS). ROS was previously considered a byproduct that impairs plant growth. However, in recent studies, ROS gained attention for its function as a signaling molecule when plants respond to environmental stresses such as heat stress. In particular, ROS, produced in response to heat stress in various plant cell compartments such as mitochondria and chloroplasts, plays a crucial role as a signaling molecule that promotes plant growth and triggers subsequent downstream reactions. Therefore, this review aims to address the latest research trends and understandings, focusing on the function and role of ROS in responding and adapting plants to heat stress.

## 1. Introduction

Plants have a sessile lifestyle that makes them very vulnerable to changes in their surroundings. One of these environmental changes is the rise in temperature caused by rapid climate change. According to the global climate change projections, the temperature will continue to rise every year, and the global average temperature in 2100 is expected to rise 1.5–2 °C above the global surface average temperature in the early 2000s [1]. Trends in crop production and changes in temperature during the growing season are closely linked. For example, crop yields are projected to continue to decline in the 21st century as global temperatures rise (i.e., 31–50% reduction in corn, wheat, and rice production) [2]. This decline in crop production threatens global food security due to the growing demand for food from a growing world population [3,4].

An increase in temperature around the plant above the optimum has a significant negative impact on plant growth. These adverse effects include a decrease in seed germination, fruit-set, pollen, and a decrease in yield [5,6,7,8,9]. The impediment to plant development is even more severe when the temperature rise is accompanied by other environmental stressors such as drought and pathogen infection [5,7,10]. In response to these harmful stresses, signaling molecules such as Ca^2+^ and reactive oxygen species (ROS) accumulate in plants, as well as alter the intracellular levels of the phytohormones such as salicylic acid (SA) and jasmonic acid (JA), which are also associated with heat stress and defense [10,11,12,13,14,15,16,17].

Previously, the accumulation of different types of ROS (i.e., singlet oxygen (^1^O_2_), superoxide (O_2_^−^), hydrogen peroxide (H_2_O_2_), and hydroxyl radicals (OH^−^)) beyond plants’ antioxidant capacity was generally believed to be highly toxic and to negatively alter the growth of plants. The accumulation of ROS, produced in response to various stresses, occurs in multiple cellular compartments, such as the cell wall, cytosol, plasma membrane, chloroplasts, mitochondria, and peroxisomes [18]. It has been reported that excessive ROS accumulation leads to lipid peroxidation, DNA damage, protein oxidation, and cell apoptosis [18,19,20,21]. Recently, ROS has gained attention as a signaling molecule that confers plants to acclimate and adapt to abiotic and biotic stresses [12,15,22,23]. Much research has been conducted to understand the dynamics of ROS effects during stress responses. Nevertheless, the understanding of the role of ROS as signaling molecules in plants during heat stress responses is still an interesting research topic. These recent studies include expression analysis of genes altered by heat stress or heat+another stress combination (i.e., heat stress+light stress, heat stress+drought stress, heat stress+pathogen infection), responses to heat stress of mutants in which genes known to be involved in heat stresses are knocked out, and direct ROS measurements under heat stress and a stress combination. The studies using transcriptomics and various genetic tools not only allow us to identify molecular targets that play an essential role in plants’ responses to heat stress accompanied by a different stress combination but also provide the necessary information to develop heat stress-resistant crops through molecular breeding. Therefore, this review aims to provide insights into the function and role of ROS on how plants utilize ROS to regulate their adaptation to heat stress and another stress combination.

## 2. The Role of ROS in Plant Development and Its Relationship to Heat Stress

ROS production occurs in plants under natural and stressful conditions both by enzymatic and nonenzymatic pathways and are associated with multiple stages of plant development and growth. Increased activity of the mitochondria, peroxisomes, lipoxygenases, NADPH oxidase, and Cytochrome P450 contribute to increased ROS production. However, environmental toxins including, UV, radiation, and heat, increase ROS production, nonenzymatically. The decrease in the activity of enzymes associated with antioxidant activity, particularly catalase (CAT), superoxide dismutase (SOD), and glutathione peroxidases (GXP) also contributes to the increased accumulation of ROS in plant cells [20,24]. ROS is essential for most aspects of plant development, including cell wall remodeling, seed germination, root growth, cell expansion, stomatal behavior, fertilization, and fruit development [25,26,27]. Apoplastic ROS, particularly the highly reactive OH^−^, interacts with cell wall polysaccharides in rapid-growing tissues such as germinating seeds and elongating radicles. Fruit ripening and ROS have developed a feedback mechanism in which the ripening of the fruit is dependent on ROS regulation [28]. For example, ROS accumulation occurs during fruit ripening and the accumulated ROS causes oxidation, altering protein function and activity, to the fruit cells that accelerate fruit ripening [29,30,31]. Stomatal behavior, opening and closing, is also regulated by ROS. NADPH oxidase enzymes such as AtRbohD and AtRbohF act to close the stomatal pores by promoting the production of ROS as a result of their interaction with Ca^2+^-Interacting Protein Kinases (CIPK), including CIPK26. Increasing antioxidants such as flavonols in guard cells lowers the ROS concentration generated, resulting in the opening of the stomata pores. Therefore, the regulation of ROS homeostasis in stomata is closely related to the regulation of the opening and closure of stomata [31,32,33]. The interaction between the apoplastic ROS and the cell wall polysaccharides results in rapid oxidation of the cell wall polysaccharides and cell wall loosening, allowing plant cells to rapidly expand cell wall during seed germination and tip growth [34,35]. Moreover, it has been shown that apoplastic ROS homeostasis during leaf expansion in Arabidopsis is regulated by the specific transcription factor *KUODA1* (*KUA1*). Expressing KUA1 during leaf growth and cell expansion increases apoplastic ROS by suppressing peroxidase activity [36]. The integrity and remodeling of the cell wall are also critical in plants’ thermal tolerance [37]. For example, *Agrostis scabra*, a heat-resistant common grass species, increases the expression of the cell wall binding protein Expansin 1 (AsEXP1) under heat stress, indicating that EXP1 functions in cell wall modification in response to heat stress [38]. It is evident that when *Poa pratensis* EXP1 (*PpEXP1*), a common meadow-grass, was overexpressed in tobacco, transgenic tobacco plants showed improved heat tolerance [39]. EXP1 induces loosening of the cell wall by weakening the bonding of the cell wall polysaccharides during cell wall reshaping. This mechanism is very similar to how ROS interacts with cell wall polysaccharides to influence cell wall looseness. However, it should be further investigated whether the increase in ROS and the expression of EXP1 in the apoplast are correlated under heat stress.

### 2.1. ROS Involvement in Seed Germination

Seed germination is the first event in the development and growth of plants, and various and complex physiological changes such as cell wall reshaping, cell expansion, and rapid tip growth occur during seed germination. In seed germination, ROS accumulation varies depending on the developmental stage of the seed. The accumulation of ROS in seeds is generally attributed to the mitochondrial activity, fatty acid oxidation of glyoxisomes, and the activity of NADPH oxidase in the plasma membrane and peroxisomes [40,41,42,43,44,45]. Accumulation of ROS during seed hydration and germination, especially H_2_O_2_ and O_2_^−^, is usually due to the continuity of mitochondrial activity [41]. The electron leakage through the Electron Transport Chain (ETC) caused by respiration occurring in the mitochondria increases free electron accumulation. These electrons can reduce O_2_, resulting in the source of mitochondrial ROS [46]. ROS above the threshold level can react with nucleic acids, affecting the genetic code of the plant. Altered ROS-induced gene expression can lead to lipid peroxidation and proteolysis, negatively affecting seed germination and viability [47]. Due to this threshold, ROS accumulation needs to be tightly controlled to have successful seed germination. Phytohormones such as abscisic acid (ABA) and gibberellic acid (GA) play a critical role in germinating seeds and developing plant growth. The production of these phytohormones are very closely linked to the temperature change during seed germination. The relationship between heat stress and these plant hormones has been reported in rice and tobacco crops. In the case of rice, GA biosynthesis was upregulated in seeds germinated under heat stress. Increased GA in seeds germinated under heat stress conditions improved seed germination by inhibiting ABA biosynthesis [9]. In tobacco, when heat stress was applied during seed germination, the efficiency of seed germination increased due to H_2_O_2_ and GA increased in germinated seeds [48].

A positive interaction between increased ROS and GA during seed germination under heat stress has been observed. Knowing the efficiency of seed germination by ROS and GA concentration and ratio under heat stress is also considered a fascinating research topic. In a germination study of cotton seeds exposed to varying temperature conditions between 4 ℃ and 44 ℃, the seeds responded positively to warm temperatures between 20 ℃ and 36 ℃ [49]. In this study, it was shown that the germination efficacy of cotton seeds is positively correlated with the expression of a specific Heat Shock Protein 24.7 (GhHSP24.7) and an increase in temperature. Heat shock proteins (HSP) are important molecular chaperones that help proteins cope with stress, more specifically heat stress [50]. Exposure to high heat for short period of time causes a burst of H_2_O_2_, this burst is then sensed by heat shock transcription factors (Hsfs). Hsfs deliver the signal to the cell and activates HSP gene expression, which have been linked to heat stress-related immune responses in plants [51,52]. As the expression of GhHSP24.7 was increased, ROS such as H_2_O_2_ and O_2_^−^ that promote germination of seeds under heat stress also increased. This accumulated evidence suggests that ROS is involved in cell expansion and cell wall reshaping in response to heat stress. ROS also acts as a signaling molecule through highly complex mechanisms, heat-sensing HSPs, and phytohormones such as GA and ABA during seed germination under the heat stress condition.

### 2.2. ROS-Mediated Root Hair Cell Expansion and Pollen Tube Growth

ROS also regulates root hair cell expansion, pollen tube growth, defense against pathogens, and initiates immune responses to abiotic stresses such as heat stress [53,54]. For tip growth and cell expansion, ROS accumulation (i.e., H_2_O_2_, O_2_^−^, lipid peroxides) due to heat stress plays a dual role depending on the type of tissue and organ and the cellular ROS threshold regulated by antioxidants such as glutathione (GSH), ascorbic acid (AsA), and flavonols [55,56,57]. For example, in Arabidopsis, ROS accumulation due to heat stress caused ferrotopsis-like regulated apoptosis (RCD) only in root cells (i.e., root hairs). However, other tissues, such as vascular and reproductive organs, did not experience RCD, although ROS accumulation occurred in response to heat stress [55]. It has been reported that heat stress applied to Arabidopsis during reproductive organ development and pollination inhibits reproductive organs’ growth, such as the stamen. However, in the stigmatic papillae, O_2_^−^ accumulation occurs due to heat stress, thereby promoting cell elongation [56]. The cell elongation caused by increased ROS in the papillae increases the distance between the stamens and the papillae under heat stress, which harms the efficiency of fertilization. In tomato pollen tubes, when the level of ROS (i.e., H_2_O_2_) is low, it plays a role in promoting the growth of pollen tubes. On the other hand, the ROS concentration in the pollen tube increases as a result of heat stress, inhibiting the growth of the pollen tube [58,59]. This may indicate that during the reproductive process, ROS signals play a role in modulating male-female interactions in response to heat stress. A study using tomato pollen tubes also reported that the increased concentration of ROS in the pollen tube under heat stress decreased ROS concentration as the synthesis of the antioxidant flavonols increased, and the growth of the pollen tube was restored and protected from heat stress [57]. Therefore, this result, which correlates with the production and accumulation of ROS under heat stress and the synthesis and increase of antioxidants such as flavonols, provides a significant clue to understanding how plants regulate ROS homeostasis and adapt to environmental stresses such as heat. The ROS signal has a dual role according to the type of tissue or organ of plants under heat stress and also shows a correlation that the ROS homeostasis is regulated according to the time and location of antioxidant synthesis. Therefore, to precisely understand the role and function of ROS in the growth and development of plant cells under heat stress, it is necessary to be aware of the characteristics of the plant tissue cells being studied and approach the study with caution.

Similar to the ROS pathway, Nitric Oxide (NO) is a vital signaling molecule that aids in many biological processes in plants including seed germination, stomatal behavior, response to abiotic and biotic stresses, and cell death. However, NO has antioxidant capabilities that are able to scavenge ROS radicals, such as O_2_^−^, at low levels [60,61,62]. Accumulation of NO has been observed in several studies, specifically for abiotic stress, like heat stress. Angiosperm stigmas, particularly *Arabidopsis Thaliana* and *Senecio Squalidus,* have been known to have increased levels of peroxidase activity when they are being pollinated. The function of the peroxidase activity was investigated in a study focusing on the stigmas and pollen of these angiosperms [63]. Concentrations of ROS, specifically H_2_O_2_, and NO were analyzed to characterize their peroxidase activity function. The findings of this study suggested that H_2_O_2_ is constitutively expressed in the stigmas of angiosperms. Interestingly, the ROS was accumulated at higher levels in the stigmas of *S. Squalidus* than in Arabidopsis. NO accumulation was increased in the stigmas of Arabidopsis than in *S. Squalidus.* The most significant finding was that the stigmas treated with endogenous sodium nitroprusside (SNP), a light-dependent NO generator, had reduced amounts of ROS accumulation in both angiosperm species. From this finding, lower ROS accumulation in the presence of NO-treated pollen is characterized as a defense mechanism against ROS at toxic levels [62,63]. Understanding the relationship between ROS and NO could be used to develop high-powered crops that have defenses against ROS-producing stresses, such as heat stress, drought stress, pathogen attack, etc.

## 3. Sites of ROS Generation in Plants under Heat Stress and Stress Combination

The natural production of ROS has various effects on the development and growth of plants. However, recently, plants have been shown to use ROS as a defense mechanism against stresses. ROS was considered a highly toxic molecule in its early discovery, and overproduction of ROS was strictly known to cause damage to the cell membrane, oxidize proteins, and reduce enzymatic activities. Excessive amounts of ROS can cause severe intracellular damage, such as lipid oxidation and single-strand breaks in DNA that cause the addition or removal of nucleotides. Plant DNA modification is induced by increased ROS levels which can lead to changes in protein structure and function that can be lethal to plants [18]. These consequences often occur when ROS production exceeds the concentration threshold in plant cells. High heat (heat stress), salinity, drought, and light are among the most common abiotic stressors in plants that contribute to the rapid accumulation of ROS when they occur independently or in combination. To better understand ROS accumulation and production effects in plants, it is critical to discuss ROS’s production sites and the effects that heat stress has on those sites. Generation of ROS occurs in various cellular compartments, including the cell wall, chloroplast, mitochondria, and peroxisomes [45]. The production of ROS causes programmed cell death induced by calcium influx in the plasma membrane. ROS causes root hair cell expansion in the plasma membrane, pollen tube growth, defense against pathogens, and initiates immune responses to abiotic stresses, including heat stress. The production of ROS in peroxisomes plays an essential role in seed and pollen germination and fruit ripening. However, the two organelles most commonly discussed concerning ROS production and accumulation in response to environmental stress, including heat stress, are mitochondria and chloroplasts, which are the main focus of this review.

### 3.1. Mitochondrial ROS Generation

The mitochondria are a vital organelle involved in the production of ROS within plant cells. The activity of the Mitochondrial Electron Transport Chain (ETC) during cellular respiration leads to electron leakage. The electrons’ free energy reduces O_2_ to ROS radicals, O_2_^−^, or H_2_O_2_ [46]. ROS accumulation aids in programmed cell death (PCD) and hyperpolarization of the mitochondria after cytochrome C release. A study done on winter wheat cells suggested that heat stress application increases the mitochondria and ROS production activity. The determination of cell viability under different heat treatments was done by treating wheat cells at 37 to 60 ℃ for 10 to 30 min. The cells were then treated with fluorescein diacetate (FDA, a cell-permeable probe for checking cell viability) and propidium iodide (PI, a fluorescent probe that can be used for staining cell wall of living cells or nucleic acids of dead cells) to determine the number of dead cells resulting from heat stress-induced ROS accumulation. Heat stress treatment from 37 ℃ to 45 ℃ showed no effect on cell viability. However, exposure for 30 min at higher temperatures, 50 ℃ to 60 ℃, led to almost complete cell death aided by protein degradation [64]. Increased cellular apoptosis is likely related to the increased mitochondrial ROS accumulation induced by heat stress to toxic and lethal levels.

### 3.2. Production of ROS in the Chloroplast

Similar to the mitochondria, chloroplasts are involved in ROS production and accumulation through photosynthetic electron transport. ROS production in the chlorophyll can be induced by abiotic stressors such as heat and light stress. When extreme light stress is exhibited on plants, photoinhibition driven by their uptake of energy from the light increases ROS production in the chlorophyll. High amounts of ROS can lead to oxidative damage to the Photosystem II complex of photosynthesis. Excess energy is absorbed in the chlorophyll, and singlet chlorophyll can be converted to deleterious triplet chlorophyll, which transfers energy to oxygen forming, ROS radical, singlet oxygen (^1^O_2_) [65]. When PSII is exposed to heat stress, heat inactivation occurs on both the PSII electron acceptor and donor sides. On the donor side, it is the cause of the inhibition of water oxidation. On the electron acceptor side, it is linked to electron transport inhibition [66]. The exposure of PSII membranes to heat stress elevates the levels of H_2_O_2_ and OH^−^. ^1^O_2_ and H_2_O_2_ play an essential role in PCD signaling under high temperatures [67].

In another study, the recovery of PSII under high light and heat stress combination (high light+heat stress) conditions were tested [68]. Plants that were subjected to both high light+heat stress in combination could not recover PSII function completely. Their survival rate was decreased to about 75%. When transcriptomic data were examined, hormonal and ROS transcripts were more prevalent in response to high light and heat stress [68,69]. Structurally, plants exposed to high light+heat stress had structural changes that increased starch granule, causing 75% of them to be distorted. However, ROS production in the chloroplasts can also be responsible for local and systemic signaling to develop systemic acclimation or resistance. In the same study, Jasmonic Acid-Isoleucine (JA-Ile) was analyzed and showed a significant increase in JA-Ile precursors. JA-Ile is a phytohormone JA conjugate with isoleucine (Ile) that functions as a signaling molecule in response to ROS over accumulation under heat stress and can be further studied to develop thermotolerant plants by fighting against the excessive ROS buildup in the chloroplast. Similarly, heat stress and high light combination, drought and heat stress affects development, yield and osmolyte accumulation in Maize [7]. The performance of two different hybrids, Xida319 and Xida889, are responsible for Maize yield production and development. Mutants Xida319 and Xida889 were exposed to a stress combination, heat+drought, over 15 days. Analysis of these mutants under this stress combination suggested that the mutant Xida889 had greater tolerance than Xida319. Increased tolerance of Xida889 was attributed to improved antioxidant activity, osmolyte accumulation, nutrient uptake, and photosynthetic pigment maintenance. Increased antioxidant capabilities in mutant Xida889 suggest that ROS accumulation is tightly controlled, which can be used to develop thermotolerant crops in the future.

## 4. Heat Stress-Induced ROS Production Aids in Plant Acclimation to Heat Stress and Stress Combination

Acclimation to an environment packed with abiotic stress stimuli is a critical system for plants to develop. Once a stress stimulus (i.e., heat and drought stress) is exhibited, plants’ systemic tissues receive the signal and begin initiating an acclimation process termed systemic acquired acclimation (SAA). SAA is a defense mechanism developed by plants that aids in preventing damage to the whole plant and induces ROS signaling transduction between different tissues in the plant [70]. In a study conducted by Zandalinas et al. [68], local and systemic response to co-occurring abiotic stresses, high light and heat stress in Arabidopsis was examined. The transcriptomic analysis revealed a significant upregulation in ROS and hormone transcripts induced by high light, or heat stress, and for both high light+heat stress when applied to separate leaves simultaneously on the same plant. The transcript results showed that signals generated from the two different leaves exposed to high light or heat stress (the combination of high light+heat stress) could communicate by sending stress-specific signals such as ROS and salicylic acid (SA, a phytohormone that plays a role in plant defense in response to abiotic and biotic stresses) accumulation, affecting gene expression. The plants exposed to high light+heat stress locally on the same leaf had difficulty expressing different signaling transcripts, acclimation to stress, and ROS waves were not as quick or visible. The results from this study suggested the application of high light+heat stress, in combination on the same leaf, initiated systemic signaling, but SAA was suppressed, suggesting that ROS and other signaling pathways in plants may be regulated differently by the stresses that the plants are perceiving. Therefore, caution must be taken when studying the response of plants to multiple stress combination.

## 5. Effects on Plant Immunity under Pathogen Attack and Mild Heat Stress

Similar to heat stress, plant pathogen attack has been identified as one of the leading causes of crop loss [71]. Understanding the relationship between heat stress and plant immunity has been a topic of interest in the plant world. In a study, Arabidopsis Thaliana plants were infected with *Pseudomonas syringae pv. tomato* DC300 (Pst DC3000) to study plant-pathogen interactions under mild heat stress at 30 °C [10]. These conditions were excellent example for stress combination, heat stress+pathogen attack. The findings of this experiment suggested that heat enhanced bacterial secretion following translocation evaluation of bacterial effector proteins, Pst DC3000 [10,72]. Pathogen susceptibility was increased, about a 2- to 4-fold difference, under mild heat stress conditions. Plant defense against pathogens or bacterial pathogen effectors are mediated by SA [73]. Due to increased pathogen susceptibility, the salicylic acid (SA) pathway was investigated under the combination of mild stress and pathogen attack in wild-type and *ics1* mutant plants. The Arabidopsis mutant *ics1* inhibits SA biosynthesis. Significant suppression of SA activity, 2-fold difference, was observed in *ics1* under mild heat stress and pathogen attack. However, the temperature difference in WT plants showed no significant affects to SA pathway activity [10,72]. The study focused on pathogen susceptibility under mild heat stress in plants, it would be interesting to investigate the same conditions under more severe heat stress. Increased temperature could cause the plants to become more susceptible to pathogen attack, lowering plant immunity defenses in both WT and isc1 mutants. However, it is important to note that this study gives an excellent example of plant-pathogen interactions induced by mild heat stress and effects on plant immune defense pathways under heat stress+pathogen attack stress combination.

## 6. The Role of ROS-Scavenging Enzymes under Thermal Stress

The ROS pathway is equipped with several ROS-producing and ROS-scavenging pathways depending on the subcellular compartments that help control ROS accumulation [53,54,74,75]. The generation of ROS at various concentrations within individual cellular compartments leads to the creation of ROS signatures. These ROS signatures help differentiate the accumulation of ROS from each cellular compartment and change depending on developmental stages or stress levels. Metabolic and signaling ROS have different functions and contribute significantly to the ROS signatures assigned to each cellular compartment. Metabolic ROS is responsible for controlling metabolic fluxes in the cell by directly altering the redox status of rate-limiting enzymes, ultimately altering metabolic reactions to counteract stress [76]. On the other hand, signaling ROS is generated as a response to stress using stress sensors and are mediated by calcium and NADPH oxidases (i.e., RBOHs) in the plasma membrane. Signaling ROS is also known to alter the redox state of regulatory proteins, translation, and transcription that activates defense responses to combat stress. ROS is also known to cause reversible and irreversible modifications to proteins, including protein degradation, fundamental in understanding how ROS can modify metabolism and gene expression during abiotic stress combination.

The most significant example given in a study was done on mitogen-activated protein kinase (MAPK) cascades. MAPK cascades are a part of signal transduction involved in plant development, hormone regulation, disease resistance, and stress responses [77]. Specifically, tomato MAK3 (SIMAPK3) has been known to defend against abiotic and biotic stresses. In tomato plants, knockout (KO) in *SIMAPK3* was compared to WT tomato plants. *slmapk3* KO mutants under heat stress had a higher survival rate than WT plants when exposed to high temperatures of 42 ℃ for 24 hours. Phenotypically, WT showed higher heat stress symptoms resulting in wilting leaves and bent stems than *slmapk3* did. Monodehydroascorbate (MDA) content and ion leakages were increased in WT plants compared to *slmapk3* mutants, suggesting more heat stress-induced damage to the cell membrane of WT plants. The most important take away from this study was that *slmapk3* KO mutants reduced the overproduction of ROS under heat stress; thus, less H_2_O_2_ and O_2_^−^ was accumulated with the help of antioxidants like superoxide dismutase (SOD), ascorbate peroxidase (APX), and catalase (CAT). SOD, APX, and CAT are plant antioxidants that are responsible for the scavenging of ROS. These enzymes can reduce H_2_O_2_, thus contributing to the development of heat tolerance.

To determine the role that antioxidants have under various thermal stresses, heat and cold, antioxidant enzyme activity for SOD, CAT, and GR, in Brassica has been studied [78]. Plants placed under cold (12 °C) and heat (32 °C) stress exhibited higher antioxidant enzyme activity than the control group kept at 20 °C. Specifically, the activity of CAT and SOD under both thermal stress conditions was increased. SOD is usually identified as the first line of defense against oxidative stress, and its function is to catalyze O_2_^−^ into O_2_ and H_2_O_2_. The H_2_O_2_ can be further reduced to H_2_O by APX to decrease its toxicity for the cell. Further analysis of the relationship between antioxidant defenses, Chlorophyll (Chl a and b) content, and leaf yield in *Brassica oleracea* crops under thermal stresses were conducted [78]. At 32 °C, phenolic content increased significantly in *Brassica oleracea capitata* varieties compared to the control group. These results suggested that plants accumulate phenolic compound content as a response to thermal stress by regulating the anabolism and catabolism of these metabolites. Chlorophyll, Chl*a* and Chl*b*, concentrations in the seedlings of *Brassica oleracea acephala* and *Brassica oleracea capitata* under 32 °C resulted in increased Chl*b* content in Capitata than in Acephala. The findings are correlated to the facilitated degradation of Chl*b*. The study concluded that Acephala is less resilient to thermal stress than Capitata. The findings contribute to the phenomenon that ROS-scavenging enzymes have a crucial role in preventing oxidative damage under a combination of abiotic stresses [54]. Previous research has focused on gene expression and enzymatic activity to understand the role of ROS involved in heat stress response. However, the magnitude that ROS affects enzymatic activity and gene expression has yet to be determined; this could lead to future research in the developing world of ROS signaling.

## 7. Conclusions

The evolving environment that plants are subjected to throughout their life span requires them to develop an adaptive mechanism to survive. As the Earth continues to warm, 1–2 °C by the year 2100 [1], plants will experience severe heat stress. Plants use intracellular signaling systems, such as reactive oxygen species, to recognize heat stress. In previous studies, the accumulation of intracellular ROS was mainly categorized as a highly toxic byproduct of essential cellular functions. Excessive ROS has been closely linked to protein degradation, lipid peroxidation, DNA damage, and cell apoptosis [18,19,20,21]. However, ROS has gained traction as a vital signaling molecule contributing to plant acclimation in response to abiotic stresses, like heat stress.

**Figure 1:**
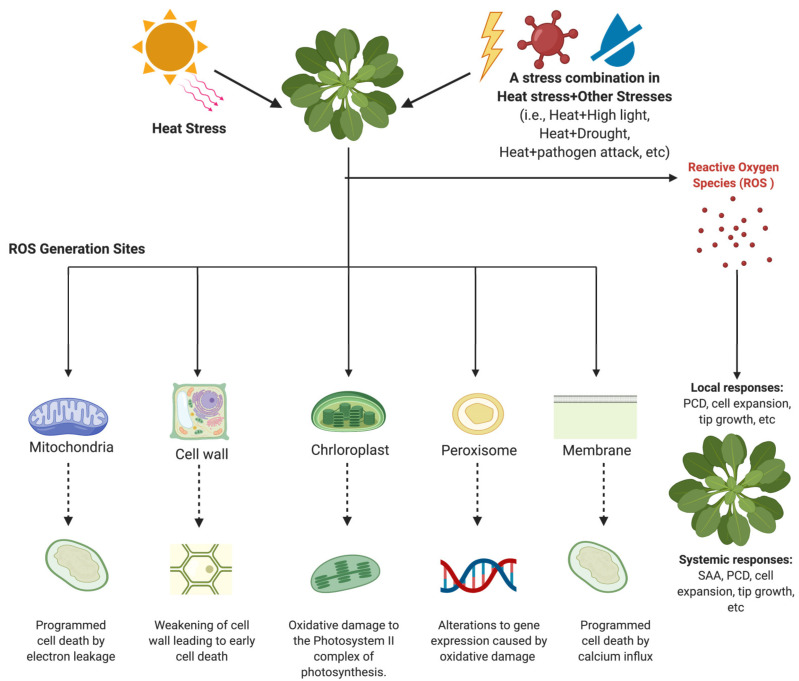
Overview of the role of reactive oxygen species (ROS) generated in response to heat or a combination of stresses (high light, drought, pathogen attack, etc.). The diagram was created using BioRender (https://biorender.com, accessed on 22 February 2021).

Several studies have found out that the accumulation of ROS affects the developmental stages of plants. Antioxidant, phytohormones, and transcription factors were thoroughly analyzed in previous studies to examine how heat stress-induced ROS affects plants’ development. Analysis of cell wall remodeling, root growth, cell expansion, stomatal behavior, fertilization, and fruit development was discussed in this review. The conclusion was that ROS accumulation led to negative and positive effects on plant development (Figure 1). However, it is essential to note that the magnitude of the correlation between heat stress-induced ROS and plant development needs to be investigated further.

When plants are exposed to heat stress, ROS accumulation is initiated. However, ROS generation sites and the effects of heat stress on those sites are crucial for understanding the role of ROS within cellular compartments. Generation of ROS occurs in various cellular compartments (Figure 1). Increased levels of ROS above the expected threshold can cause PCD induced by calcium influx in the plasma membrane. Root hair cell expansion, pollen tube growth, defense against pathogens, and initiation of immune responses to stresses are also differently regulated by increased ROS levels. In the peroxisomes, ROS is essential for seed and pollen germination and fruit ripening. Two of the commonly discussed organelles involved in ROS production and accumulation in response to heat stress and stress combination in heat and other stresses are the mitochondria and the chloroplast. In these organelles, electron leakage from ETC is usually due to ROS, produced in response to heat stress. While ROS generation sites have been studied, the maximum threshold for ROS accumulation that leads to toxicity and inhibits growth should be the focus of future research.

The tight control of ROS aids in the development of acclimation mechanisms in plants to a changing environment. Once a stress stimulus (i.e., heat stress, drought stress, or stress combination) is exhibited, plants’ systemic tissues receive the signal and begin initiating SAA to prepare adaption to future possible stimuli or damages. SAA is a defense mechanism developed by plants that aids in preventing damage to the whole plant and induces ROS signaling transduction between different plant tissues [70]. Many studies suggest that the ROS pathway is equipped with several ROS-producing and ROS-scavenging mechanisms, that help control ROS accumulation and contribute to plant acclimation [75]. Gene expression and antioxidant enzyme activity have been studied closely to determine their ROS-scavenging roles, most leading to increased plant tolerance to abiotic stress combinations. The location and timing of ROS production and how it contributes to the acclimation process in plants concerning heat stress is yet to be discovered.

In order to understand how heat stress-induced ROS production initiates responses in various cellular compartments, it is critical to know the detailed dynamics of ROS production and decline. However, it is still one of the biggest challenges to detect the exact the moment of ROS production and patterns of ROS changes. These are due to several reasons, including: (1) most of the chemical ROS probes lack specificity to detect specific ROS species. For example, dichlorodihydrofluorescein diacetate (H_2_DCF-DA) is widely used to detect ROS, especially H_2_O_2_, from cellular levels to whole plant level. However, H_2_DCF-DA not only detects H_2_O_2_ but also reports other ROS species since the dye is sensitive and is oxidized by any oxygen driven ROS species. (2) Most of the fluorescence and color metric ROS probes do not have the capability of reporting ROS decrease due to the irreversible reaction of the probes after oxidation by ROS generated in response to stresses. (3) The synthetic probes are often hard to control. Specifically, targeting to the specific cellular compartments or tissues to investigate ROS dynamics is very difficult to control due to bleeding of the probe between adjacent compartments and cells. (4) Each cellular compartment has different amounts of ROS generation in response to specific stress. The challenge is choosing the correct ROS probe because the K_d_ for ROS detection of each probe is either too low or high to meet the ROS threshold required for detection in a cellular compartment. In addition, most of the fluorescent and chemical probes and ROS bioreporters are widely used for qualitative measurement of ROS production, which do not allow researchers to detect actual concentration of ROS produced. Recently, Lim et al. reported detection of ROS using newly developed ratio metric Zat12p-ROS bioreporter, which reported detection of ROS increase and decrease in response to salt and oxidative stress using ratio calculation of GFP (ROS bioreporter signal) over mCherry (internal reference fluorescence signal) signals detected in root and shoots [79]. Although this study showed that Zat12p-ROS was able to detect decreases in ROS production, the exact mechanisms of how either GFP and mCherry signals are changed upon ROS production in their experimental conditions are still unclear. Thus, caution must be taken when ROS bioreporters are used to detect changes in ROS dynamics. In addition, several ROS biosensors such as HyPer, HyPer-3, and HyPer-Red have been developed for quantitative detection of H_2_O_2_ [80,81,82,83,84,85]. While these ROS biosensors allow quantitative detection of ROS production and the changes in ROS dynamics, it is still difficult to use these biosensors because of their biochemical characteristics of ROS detection (i.e., an excitation shift in the presence and absence of ROS with single emission). Therefore, an improvement of these biosensors will be beneficial to understand ROS dynamics in response to heat or other stresses.

In this review, the role of heat stress-induced ROS in the development, production, and acclimation of plants have been explored. However, there are still areas where future research could continue—for example, the magnitude for which heat stress-induced ROS affects plant development, production sites, and plant acclimation. Further research in these areas could lead to thermotolerant crop production as the increasing temperatures from climate change continue to threaten the nation’s food security.

## Data Availability

Data sharing not applicable.
